# Exploring exchange and direct procurement strategies for Natufian food processing tools of el-Wad Terrace, Israel

**DOI:** 10.1038/s41598-021-88484-1

**Published:** 2021-05-04

**Authors:** Danny Rosenberg, Tatjana M. Gluhak, Daniel Kaufman, Reuven Yeshurun, Mina Weinstein-Evron

**Affiliations:** 1 Laboratory for Ground Stone Tools Research, The Zinman Institute of Archaeology, University of Haifa, Haifa, Israel; 2 Römisch Germanisches Zentralmuseum, Ernst Ludwig Platz 2, 55124 Mainz, Germany; 3 The Zinman Institute of Archaeology, University of Haifa, Haifa, Israel

**Keywords:** Geochemistry, Geology, Mineralogy, Petrology

## Abstract

We present the results of a detailed geochemical provenance study of 54 Natufian (ca. 15,000–11,700 cal. BP) basalt pestles from the site of el-Wad Terrace (EWT), Israel. It is the first time precise locations from where basalt raw materials were derived are provided. The results indicate that the Natufian hunter-gatherers used multiple sources of basaltic rocks, distributed over a large area surrounding the Sea of Galilee. This area is located at a considerable distance from EWT, ca. 60–120 km away, in a region where contemporaneous Natufian basecamps are few. We consider two possible models that suggest vehicles for the transportation of these artifacts to EWT, namely the exchange obtaining model (EOM) and the direct procurement model (DPM). We argue that these mechanisms are not mutually exclusive and may have operated together. We also suggest that at a time of increasing Natufian territoriality, a large area around the Sea of Galilee remained unclaimed. The paper concludes with a brief discussion of the implications for the two models. In particular, we note that the DPM implies that technological know-how for pestle production was maintained within the EWT community.

## Introduction

The nascence of sedentism profoundly impacted human societies’ mobility patterns, requiring a host of adjustments. Among its immediate implications are economic intensification and preoccupation with production and territoriality, both within and across communities. In the southern Levant, these developments are epitomized by the Natufian culture (ca. 15,000–11,700 cal. BP^1–8^), notable for its preference to intensively exploit the site’s catchment area. The site of el-Wad Terrace (EWT) in Mount Carmel (Israel) illustrates this well. Its inhabitants drew on a broad spectrum of resources, including plants^9–11^, animals^12,13^, ochre^14^ and flint^15,16^, all of which derive from within the site’s immediate vicinity. For hunted ungulates, this pattern is manifested in the comparatively complete body-part profiles, indicating that the hunt took place close by and that one did not have to haul the catch over great distances^17^. For other resources (e.g., flint, ochre, mollusks), it is estimated that they were retrieved from locations no more than 12 km away^16,18^.

Thus, the advent of a sedentary way of life dovetailed with the emergence of an early sense of possession. As groups became more closely attached to a certain place and invested in their immediate surroundings, they probably began cultivating prefatory claims of ownership^19,20^. In this vein, the Natufian culture is also notable for introducing a new sort of geopolitics: the emergence of socio-territorial entities, a landscape of more-or-less distinct spatial units attached to organic groups, probably separated from one another by unclaimed “buffer zones.” As one group claims an area and its resources, it also denies it to others, setting into motion a dialectic of alienation and suspicion. Under such circumstances, every unwarranted entry readily becomes a threat and an act of aggression^21^. Yet, while the Natufian culture marks a rise in raw material exploitation and selectivity^22,23^, increasing territoriality and decreasing mobility, the widespread distribution of basalt tools and, in particular, pestles, suggests a mechanism that encouraged outreach and mobility for obtaining these desired food processing tools. As these artifacts often derive from distant locations, they implicate procedures that span considerable distances. They entailed trade/exchange across groups, as suggested by Weinstein-Evron and colleagues^24,25^, purposeful and predetermined forays to distant sources or a combination of the two.

Variation in procurement strategies and exchange among prehistoric societies has been linked to factors such as mobility, technological design, landscape use and cultural perceptions of the landscape^26–29^. The acquisition and movement of raw materials is often discussed and understood in terms of embedded and direct procurement strategies^30^. While the specific characteristics of raw material procurement vary in details among different societies, embedded strategies pertain to the collection of raw materials in an ancillary manner while moving through a landscape for other focal purposes such as foraging. Direct procurement, on the other hand, represents specific travel to the sources of raw material or to obtain any desired goods^26,31^. While various modes of obtaining raw materials or tools may have different archaeological signatures (e.g., the number of sources used or the morphometric uniformity of the desired end product), differentiating between the various possible scenarios is not straightforward^30^.

**Figure 1:**
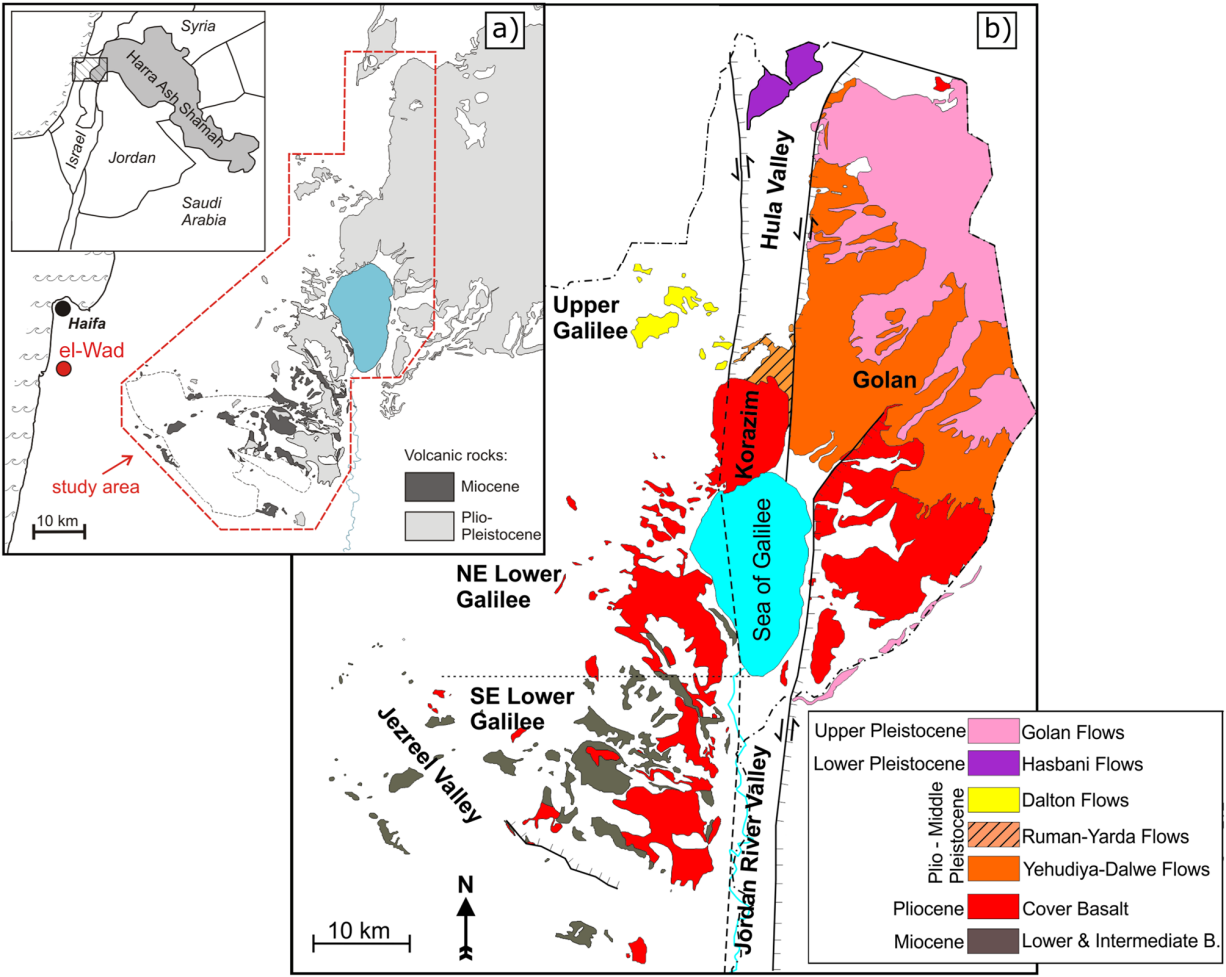
(**a**) Location map of the study area, (**b**) simplified geological map of the Neogene and Pleistocene volcanic rocks in northern Israel based on Bogoch and Sneh^33^, Hatzor^34^, Levitte and Sneh^35^, Sass et al*.*^36^, Sneh^37^, Sneh and Weinberger^38,39^ and Sneh et al.^40^ (created by T.G. in QGIS Pisa and Inkscape 0.92).

**Figure 2:**
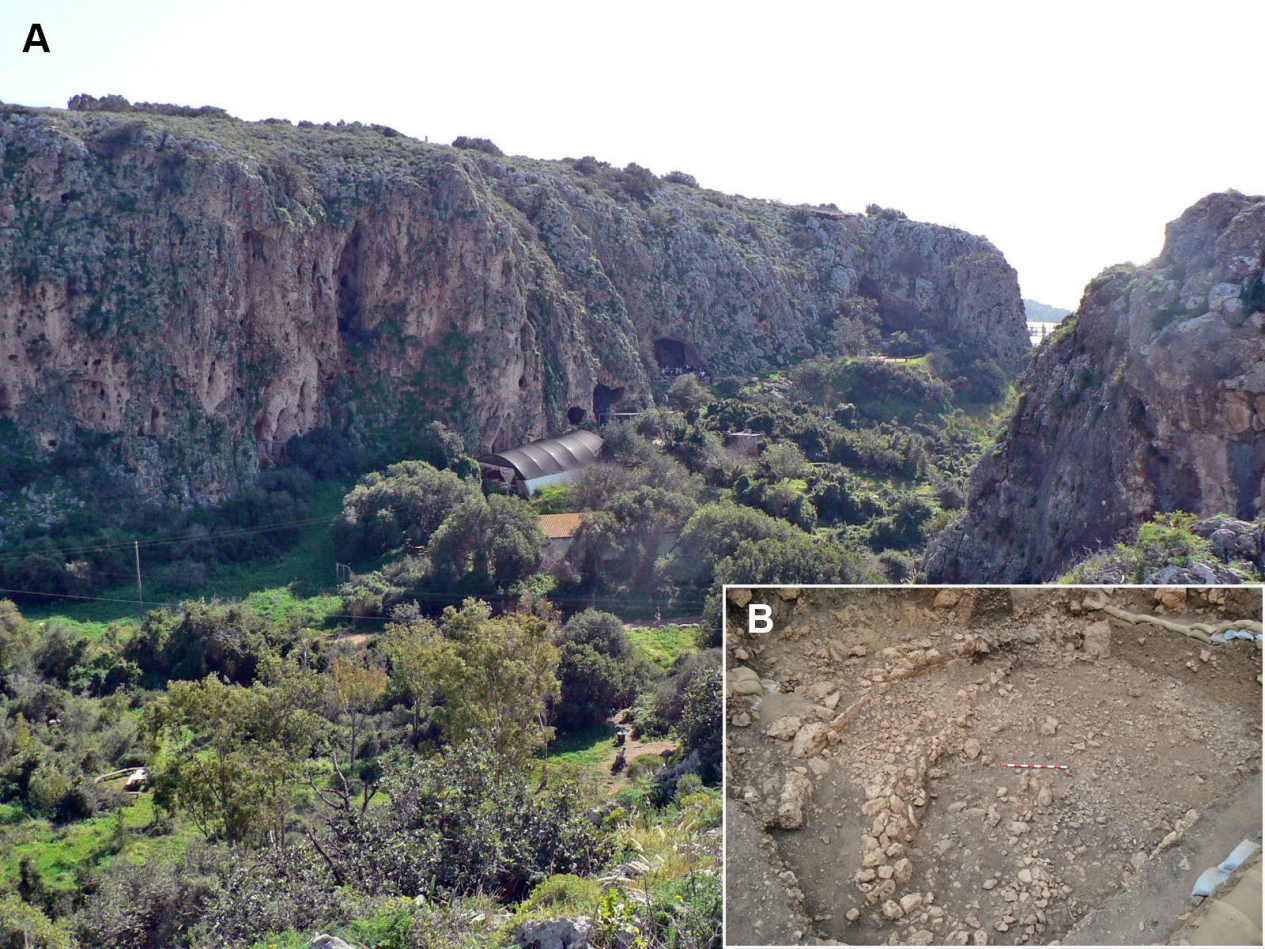
(**A**) A view of Nahal Meʽarot Caves. EWT is located under the greenhouse near the cliff; (**B**) EWT during the excavations. Note the long curvilinear “terrace wall” and enclosed/incorporated structures and living surfaces of this Natufian sedentary hamlet (for a plan, see Fig. S1. Photographs by R.Y.).

This paper presents the results of a meticulous geochemical provenance study of 54 Natufian basalt pestles from the site of EWT (Figs. 1, 2). The geochemical data of the artifacts and geological samples were collected by x-ray fluorescence and laser ablation inductively coupled mass spectrometry to determine the pestles’ geological origins by comparing them to our own field database with the aid of cluster analyses (see methods section for details). This allows discussion of the possible ways these may have been obtained by the inhabitants of EWT. Previous studies in this vein are few and, at most, of preliminary significance. Of particular relevance for our purposes is a study by Weinstein-Evron and colleagues^24,32^ of 22 basalt tools from el-Wad and other Natufian sites in the Galilee. They sought to identify the raw materials’ origins by applying K–Ar dating to the basalt items and potential geological sources. They observed that the raw materials used to manufacture these items derive from basalts dated to the Pliocene and Pleistocene. Basaltic rocks of these ages are altogether absent from Mount Carmel, but they are accessible in the Galilee, Golan Heights and various other areas east of the Jordan Valley (Fig. 1^33–40^). However, determinations that were more precise were not forthcoming.

Picking up from where Weinstein-Evron and colleagues left off, our study draws on the geochemical and mineralogical features of basaltic rocks to (1) articulate the assemblage’s compositional variability and (2) trace pestles back to the provenance from which their raw materials were procured. We argue that the EWT pestle assemblage originated from multiple geological sources. We discuss the possible mechanism of trade/exchange as a possible explanation (we call this hypothesis the Exchange Obtaining Model or EOM). In such a circumstance, the Natufian pestle producers would exploit a single or a limited number of basalt sources (likely having used a preferred raw material source suitable for pestle production^26^). It is not however the only plausible hypothesis, and we consider the alternative of direct procurement. Whallon^41^, for instance, suggested that hunter-gatherers may move and interact across mesoscale social networks, seeking access to diverse resources and exotic items. Accordingly, we can prudently suggest that the inhabitants of EWT may have made their way as far as the Golan Heights to acquire the materials for their pestles directly from the source (we call this hypothesis the Direct Procurement Model or DPM), and we predict that it will be substantiated by more diverse sources used. We discuss the implication of these two models for our understanding of the Natufian mobility pattern and control over the know-how of pestle production.

### The Natufian site of EWT and its food processing stone tools

EWT is the northeastern part of the site of el-Wad (Fig. 2; Fig. S1), one of the largest Natufian hamlets in the southern Levant. It is located near the Mediterranean coastal plain on the western face of Mount Carmel, Israel. Mount Carmel consists of sedimentary rocks—mainly limestone and dolomite—and intercalated Cretaceous volcanic rocks (Fig. 1). An area of ca. 70 m^2^ was excavated at EWT, exposing a > 1.5 m thick sequence of Natufian deposits^42,43^. A composite stratigraphy of the site^42,44,45^ suggests a sequence spanning the temporal range of ca. 15,000–12,000 cal. BP^46,47^.

Consisting of nearly 600 items, the ground stone tools assemblage of EWT^48^ is one of the largest of its kind, most found in the Early Natufian layers (nearly 60% of the assemblage). Of the various food processing tool types (Fig. S1), basalt pestles, mainly represented by small fragments (Fig. S1), are the most abundant (22.8% of the assemblage). Most of them derive from Early Natufian contexts (ca. 70% of the total pestles), and only a handful originated from Late Natufian phases or indeterminate contexts. Of these, a total of 47 Early Natufian and seven Late Natufian pestle fragments were chosen for geochemical analysis.

## Results

**Figure 3:**
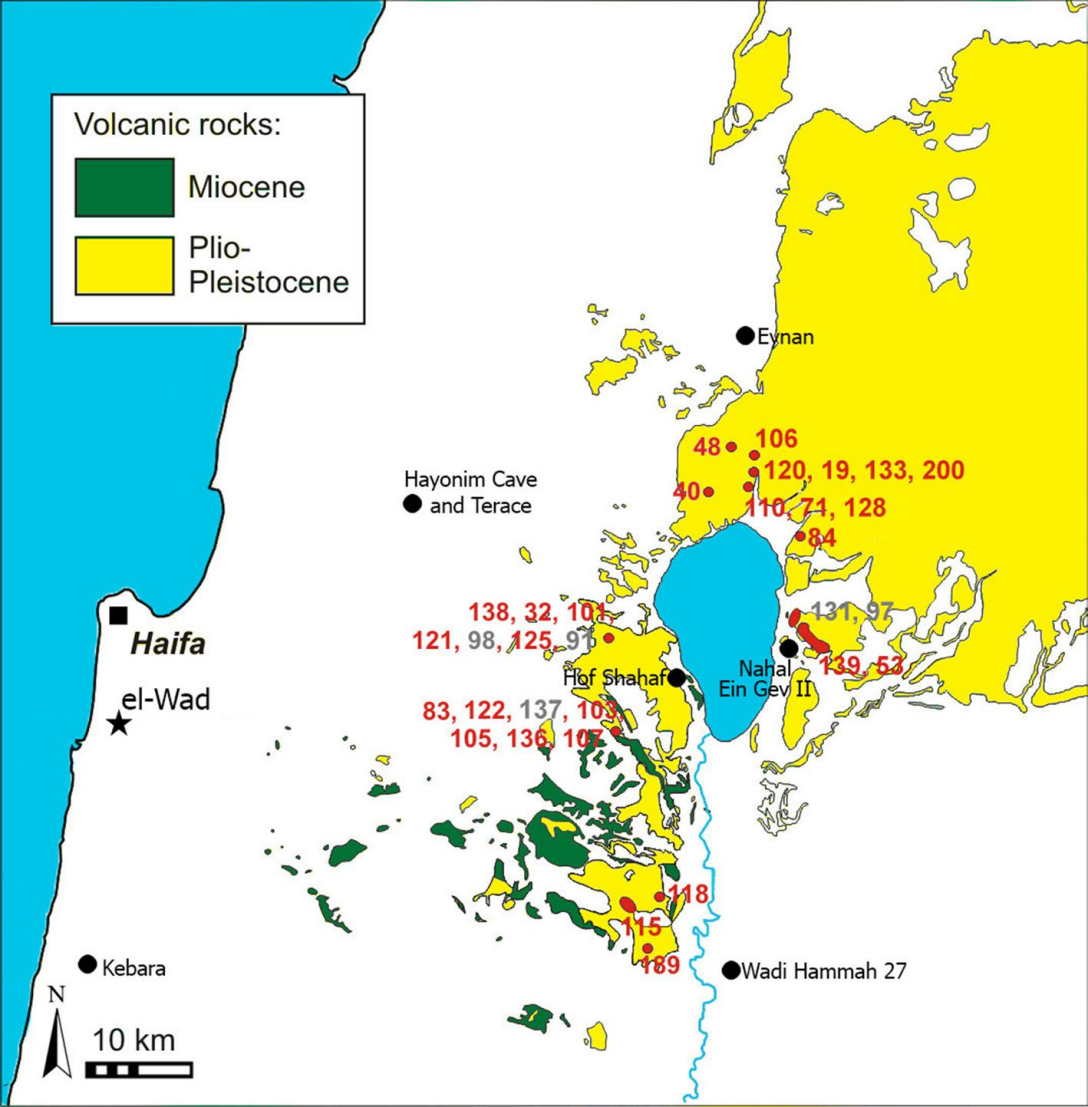
Provenance of the EWT pestles (see Table S1). Red dots indicate exact locations. The fields indicating the provenance of EWT 131 and 97, of EWT 139 and 53, and EWT 115 (marked in grey) define the area where the corresponding geological samples were collected. (created by T.G. in QGIS Pisa and Inkscape 0.92).

All pestles analyzed are of fine-grained non-porous volcanic rocks. The classification in the TAS-diagram (Fig. S2, Le Bas et al.^49^, here plotted with the geological data from Gluhak and Rosenberg^50^ and the present study) shows that the majority of the pestles are produced from alkali basalt. Two artifact samples are basanites (with more than 10% normative olivine). Five artifacts plot in the trachybasalt field; one is transitional from alkali-basalt to trachyandesite. All of the trachybasaltic samples can be defined as hawaiites. The distribution of major and trace elements (Fig. S3, Table S1) suggests a distinction of several possible sources; the distribution of rare earth elements (REE, Fig. S4) suggests a distinction between at least two groups. However, the major elements indicate greater differences in the artifacts’ geochemical compositions than the trace elements do (Fig. S4). Plotting the distributions of chemical elements derived from archaeological specimens against those of the geological samples (Fig. 3, Fig. S3, Table S2) demonstrates that the raw materials of the EWT pestles originate from the lava flows of the Cover Basalt, which are located at a substantial distance of 60–120 km from the site, scattered around the Sea of Galilee.

Cluster analyses (average linkage clustering with city block and Euclidean distance and ward clustering with Euclidean distance) of the pestle samples together with the geological samples distinguished at least 18 sources, of which 13 were traced to specific locations around the Sea of Galilee (Fig. 3, Table S1). Most analyzed pestles (44.4%) originate from the lava flows west (14 pestles) and north of the Sea of Galilee (10 pestles, the Korazim basalt block); fewer were traced to locations east (five pestles, western slopes of the Golan Heights) and south of the Sea of Galilee (three pestles, western slopes of the Jordan Valley). The provenance of the remaining twelve is not yet determined.

## Discussion

The present study is the first successful attempt to trace Natufian basalt tools to precise geological sources. Previous attempts to do so applied radiometric dating techniques and resulted in significant but, for the greater part, equivocal and indefinite results. They succeeded in narrowing the range of probable sources to general regional and geological whereabouts—Pliocene and Pleistocene basalts of the Galilee, Golan Heights and various areas east of the Jordan Valley, but they were unable to go further^24,25,32^. Our study, on the other hand, conducted a comparative high-resolution analysis of geochemical data for the tools and their potential geological sources. The results suggest that the Natufians of EWT were using pestles made of basalts that originated from multiple geological sources, all of which are located at a substantial distance of 60–120 km from the site. Specific and accurate results were provided for the majority of analyzed items (59.3% of the tested basalt pestles), all of which were shown to derive from Pliocene Cover Basalt around the Sea of Galilee, mainly to the north and west and less so to the east and south (the geographic distribution of the sources exploited spans an area of at least 1200 km^2^).

The two models that were set to explain the Natufian acquisition of basalt tools (EOM and DPM) reflect two different modes of interaction and mobility. While the EOM gained considerable traction over the past two decades, widely echoed in syntheses of the Levantine Epipaleolithic, especially those concerned with inter-group ties^51–53^, we see the other model (DPM) as equally applicable, bearing in mind hunter-gatherer mobility and interaction across social networks^41^. In either case, as noted by Kaufman^54^ and Weinstein et al.^25^, the social networks provided the foundations for the procurement of essential raw materials for pestle production. Systems of alliances and the establishment of mutually reciprocal social obligations between Natufian groups served to guarantee access to raw materials or enable trade/exchange.

Thus, deciding between the two models (EOM and DPM) for the basalt pestles of EWT is difficult and requires additional datasets from other fields, such as symbolic representations, lithics and even isotopic studies of faunal remains. In fact, it is probable that both mechanisms were at play, constituting different, perhaps complementary, channels for the circulation of substances and goods. The small number of Early Natufian base camps in the Mediterranean climatic zone and the comparatively imprecise dates available for them pose considerable obstacles for any attempt to determine relationships among groups and territories^55^. Therefore, it is questionable whether links can be confidently established between certain Natufian sites and favorable basalt outcrops. Moreover, even if the raw material was located at a reasonable distance, the lack of indications for basalt tool production at Natufian basecamps^48^ renders any association of this sort unsubstantiated and circumstantial at best. This is particularly notable for sites like Early Natufian Wadi Hammeh 27^56^, Hof Shahaf^57^ and the Late Natufian Nahal Ein Gev II^58^. At Wadi Hammeh 27, there is a rich and varied assemblage of basalt tools, four of which were geochemically tested and showed a non-local origin (probably in the southern outcrops of Wadi Mujib and near Kerak)^59^, and at Hof Shaf and Nahal Ein Gev II, there are no signs for intensive basalt production^57,58^, despite their location in the vicinity of the extraction sites established in this study.

While neither these observations nor our geochemical analyses disprove the EOM, it seems that they do lend some force to the DPM. First, they suggest a diffused and decentralized network that lacked specialized nodes for procurement, production, transport or consumption. This diffused network is implied by the wide distribution of extraction sites (Fig. 3, Table S2), the absence of major production sites and the conspicuousness of the basalt ground stone tools across all Natufian occupations. Thus, contrary to the EOM expectations, the various nodes (i.e., sites, communities) seem to have fulfilled all or most functions. Second, while directing our attention to the area surrounding the Sea of Galilee, our geochemical analysis also points out that the Natufian inhabitants of EWT chose not to use suitable high-quality basaltic rocks available in closer proximity^60,61^. As this is unlikely to have been due to economic considerations or incognizance; socio-territorial constraints are probable.

Interestingly, while the two mechanisms are not mutually exclusive and may have operated simultaneously and perhaps in combination, they have different implications and embody different priorities. Specifically, the EOM entails less mobility but more inter-group interaction, whereas the DPM entails greater mobility in space and less inter-group interaction. Thus, if the EWT inhabitants obtained their basalt for pestles via direct procurement, they were also engaged in maintenance and preservation of their technological know-how. On the other hand, if their pestles were obtained via an exchange mechanism, the know-how of pestle production was probably held by others, constituting a more restricted and controlled body of knowledge. Admittedly, our analysis does not resolve these issues. But it does offer support for a hypothesis that is yet to receive the attention it deserves, and it does demonstrate how basalt tool provenance studies have the potential to tap into constitutive ideological and behavioral features of the Natufian culture.

## Methods

### Sampling

The entire basalt food-processing tool assemblage of EWT was studied in the Laboratory for Ground Stone Tools Research (LGSTR) at the Zinman Institute of Archaeology, University of Haifa, Israel. All metric and morphological traits were recorded^48^. As pestles are the largest tool group in the assemblage (nearly 25% of the total tool count, that include small and unidentified tool fragments and over 60% of the defined tools) and as these are the most dominant tool type in most Natufian assemblages, they were a natural candidate for the current analysis. For technical sampling considerations, we chose the largest available pestle fragments (over 40% of the pestles) to minimize damage to the appearance of the artifact. Fifty-four pestles were selected for analysis: 47 from the Early Natufian assemblage (over 50% of the Early Natufian pestles) and seven from the Late Natufian assemblage (100% of the Late Natufian pestles). Samples for geochemical analysis were produced by removing a small piece with an iron chisel.

### Geochemical analyses

The generation of geochemical data for the artifacts and the analysis of these data against the comparative geological database were conducted at the Institute for Geosciences at the Johannes Gutenberg University, Mainz, Germany. Analyses concentrated on the specimens’ geochemical composition. All samples were first tested for loss on ignition (LOI). Subsequently, glass beads were produced for x-ray spectrometry on an iridium strip-heater^62^. The samples were analyzed for major elements utilizing a wavelength dispersive 2002 Philips MagXPro X-ray spectrometer. On two occasions that the samples were too small for major elements analysis by XRF, the analysis was conducted with a Jeol JXA 8900 RL electron-microprobe (EMP), with an acceleration voltage of 15 kV, a beam current of 12 nA and a beam diameter of 5 μm. Five spots were measured per sample. In order to guarantee the compatibility of the major element results across devices, the EMP results were converted into the XRF-results using the calibration presented by Gluhak and Rosenberg 2013^63^.

Trace elements were measured in an Agilent 7500 CE quadrupol ICP-MS. The samples were ablated from the glass beads by an ESI New Wave Research NWR 193 (ArF-excimer) laser ablation-system. Three spots were measured on every glass bead, with a diameter of 100 μm, a pulse rate of 10 Hz and laser densities of ca. 6 J/cm^2^. ^43^Ca measures served as internal standards, taken from the XRF-measurements and the EMPA-results. As reference material, NIST SRM 612, NIST SRM 610 and USGR BCR-2G served for quality control. The values were taken from the GeoReM online database^64^. Data reduction was carried out on GLITTER 4.4.2 software (Macquarie University, Sydney, Australia). The analyses’ results for the artifacts and their computability with specific geological samples are given in Tables S1, S2 and S3.

In order to articulate the raw material variability within the EWT pestles (i.e., to analyze how many groups of Cover Basalt rocks, each possibly representing an individual occurrence within the Cover Basalt unit, are “hidden” within this set of artifact data), cluster analyses were calculated. For this purpose, all geochemical data were log-transformed and z-standardized. They were then subjected to different cluster algorithms: Average linkage (with City Block and Euclidean distance) and Ward (with Euclidean distance). Artifact clusters were only considered when the different cluster algorithms formed consistent groupings. These clusters were defined as “virtual extraction sites” (specific, at this point, unidentified extraction sites^50,60^) where the raw material for the basalt pestles was procured. The results of the artifact clusters are presented in Table S2. Afterwards, the artifact samples were clustered with the geological field samples^50^ (and this study, see Table S3). The geological samples associated with these clusters were interpreted as their source. The provenance of single artifacts or a group of artifacts was only regarded as determined if the different cluster methods agreed on the affiliation of the same geological samples to the same artifact or cluster of artifacts. In the cases where the different cluster methods associated different geological samples in close spatial proximity to the same artifact, the respective area was defined as their provenance. All other EWT artifact samples, which were not affiliated unequivocally to certain geological samples, are individual samples whose provenance could not be determined based on the present data. The results of these cluster analyses are presented in Table S2.

### Excavations

Renewed excavations at EWT were conducted by the University of Haifa with the acquiescence of the Israel Antiquities Authority; excavation licenses G-15/2001, G-8/2002, G-13/2003, G-28/2004, G-13/2005, G-20/2006, G-3/2007, G-2/2008, G-4/2009 and G-5/2010. All stones suspected to be worked or appeared to have derived from a non-local geological milieu were kept and examined. The excavated sediments were sieved in their entirety, and even small items were recovered (i.e., small basalt flakes). All pestles discussed in this paper derive from these excavations and constitute part of the ongoing study of the site’s stone tool assemblage^48^.

## Supplementary Information

Supplementary Figures

Supplementary Tables
