# Supplementary material for: Exploring exchange and direct procurement strategies for Natufian food processing tools of el-Wad Terrace, Israel
Source: Sci Rep. 2021 May 4;11:9480. doi: 10.1038/s41598-021-88484-1 (PMC8096842; doi:10.1038/s41598-021-88484-1)
Supplement: Supplementary file 1 — Supplementary Figures [file 41598_2021_88484_MOESM1_ESM.docx]

**Supplementary data**


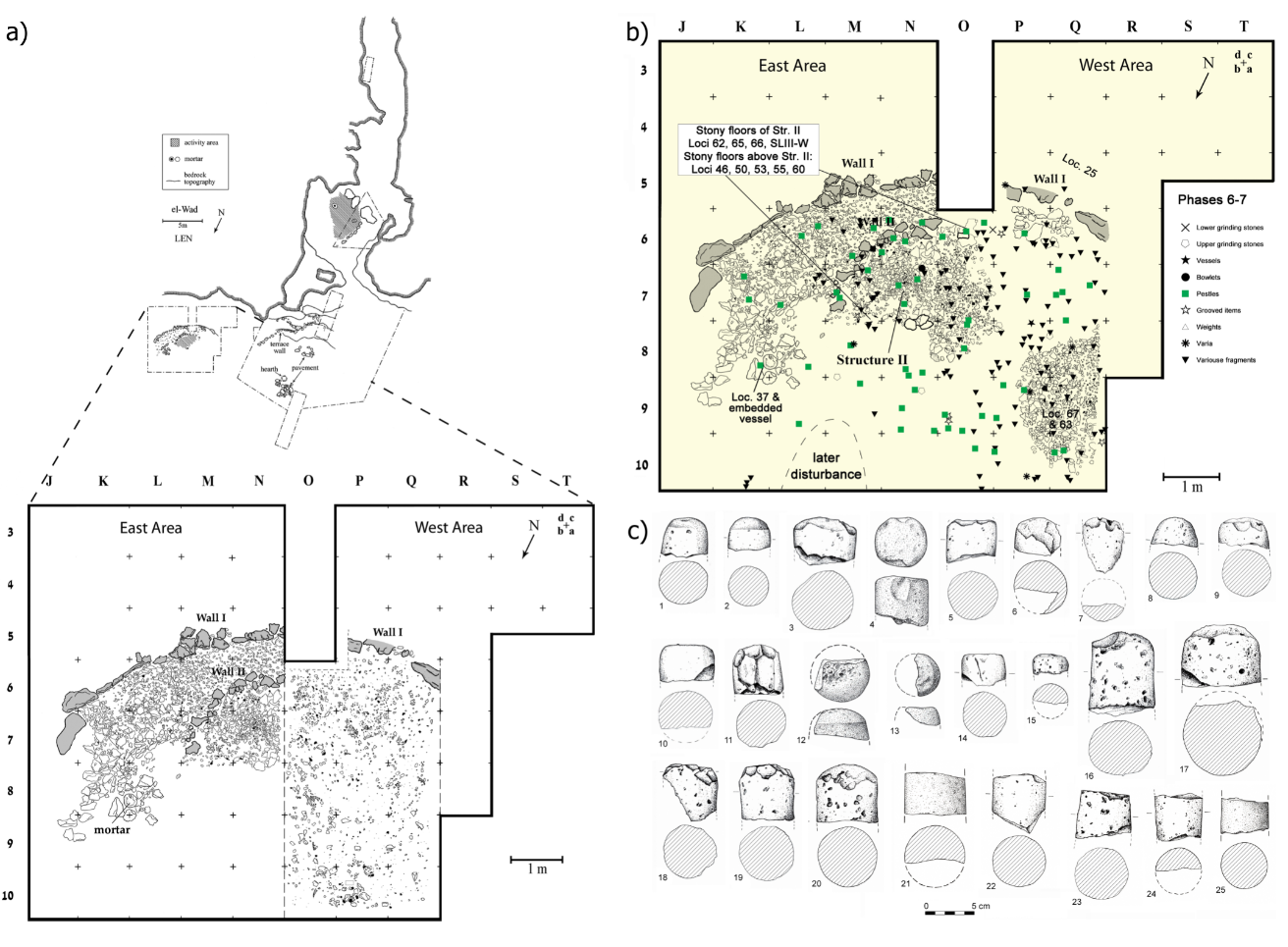


Fig. S1. a. Spatial distribution of ground stone tools in the Early Natufian phases of EWT^2^; b. Basalt pestle fragments from EWT.


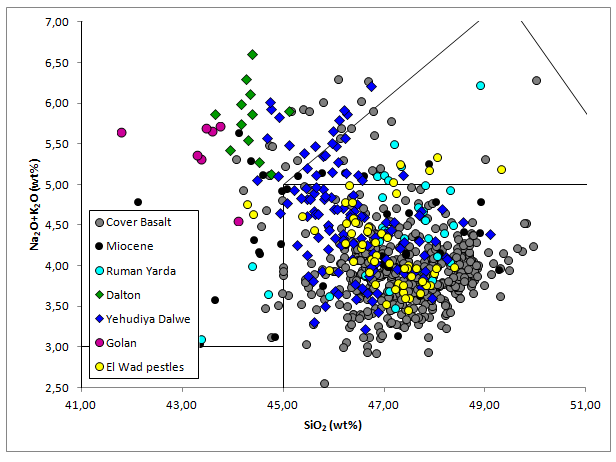


Fig. S2. Total-alkali-silica-diagram^3^ of the EWT and geological samples. Excerpt from SiO_2_ 41-51 wt% and Na2O-K2O from 2,5 to 7 wt%". (data from Gluhak and Rosenberg^1^ and this study).


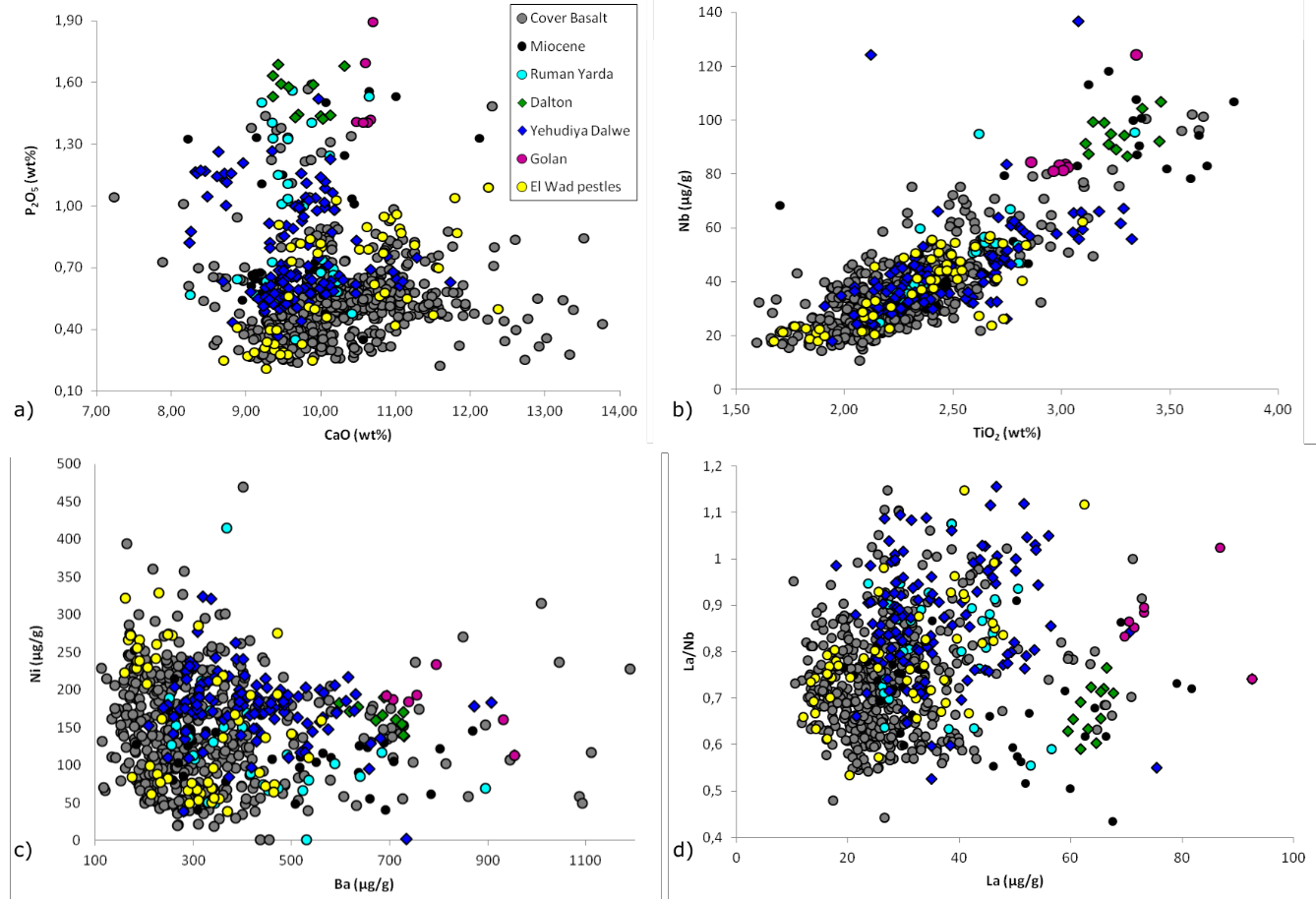


Fig. S3. Geochemical composition of the EWT samples and the geological samples; a. CaO-P2O5, b. TiO2-Nb, c. Ba-Ni; d. La-La/Nb. Data from Gluhak and Rosenberg^1^ and this study.


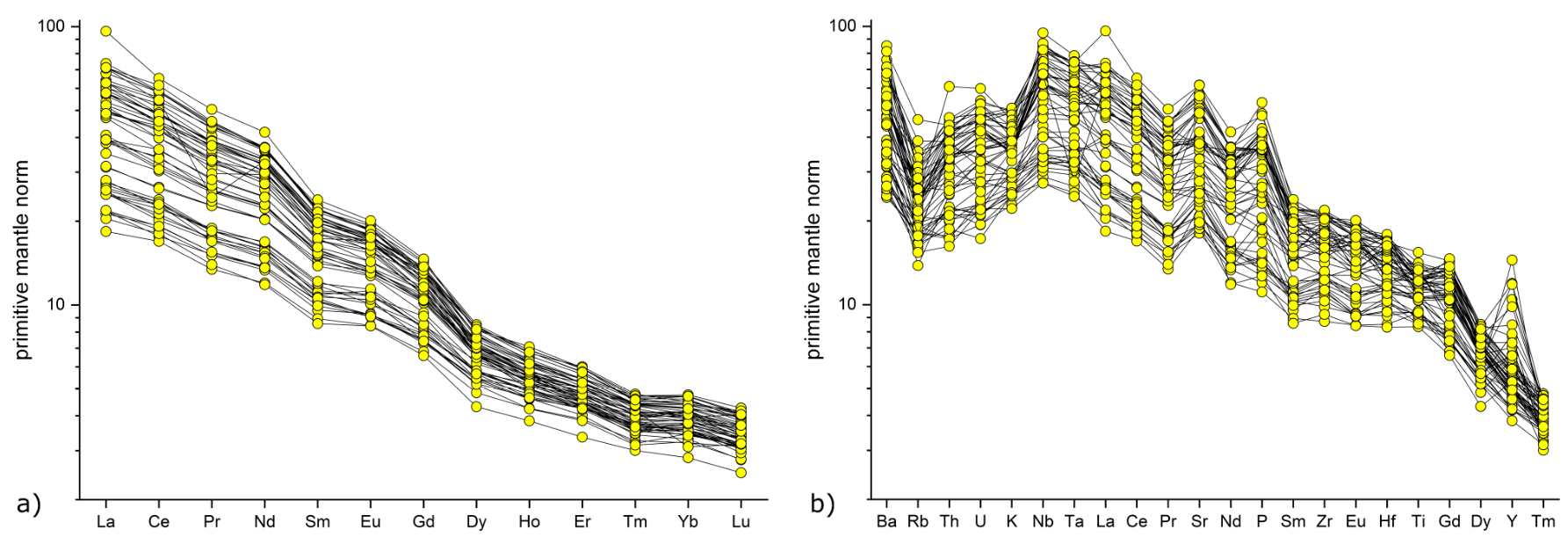


Fig. S4. Primitive mantle normalized trace element diagrams of the EWT samples; a. rare earth elements; b. incompatible trace elements. Primitive mantle composition from McDonough and Sun^4^.

**Supplementary Data - References**

1. Gluhak, T. & Rosenberg, D. Back to the source—Geochemical data from Israel for the provenance analyses of basaltic rock artefacts and their implications on previous and future studies. *Archaeometry*. **60**, 1153-1169 (2018).
2. Rosenberg, D., Kaufman, D., Yeshurun, R. & Weinstein-Evron, M. The broken record: The Natufian groundstone assemblage from el-Wad Terrace (Mount Carmel, Israel)—Attributes and their interpretation. *Journal of Eurasian Prehistory*. **9**, 93-128 (2012).
3. Le Bas, M. J., Le Maitre, R. W., Streckeisen, A. & Zanettin, B. A chemical classification of volcanic rocks based on the total alkali-silica diagram. *Journal of Petrology*. **27**, 745-750 (1986).
4. McDonough, W. F. & Sun, S.-S. The composition of the Earth. *Chemical Geology*. **120**, 223-253 (1995).
